# The zebrafish model: a versatile platform for uncovering the therapeutic potential of phytochemicals in liver diseases

**DOI:** 10.3389/fphar.2026.1794777

**Published:** 2026-04-28

**Authors:** Yuliang Liu, Xiang Meng, Wei Zhang, Mengmeng Sun, Ziyoviddin Yusupov, Komiljon Tojibaev, Kobil A. Bobokalonov, Zhenghai Zhang, Min He, Meiying Jin

**Affiliations:** 1 Changchun University of Chinese Medicine, Changchun, China; 2 The Jilin Province School-Enterprise Cooperation Technology Innovation Laboratory of Herbal Efficacy Evaluation Based on Zebrafish Model Organisms, Changchun University of Chinese Medicine, Changchun, China; 3 Changchun Stomatological Hospital, Changchun, China; 4 Institute of Botany, Academy of Sciences of Uzbekistan, Tashkent, Uzbekistan; 5 Institute of Botany, Plant Physiology and Genetics, National Academy of Tajikistan, Dushanbe, Tajikistan; 6 College of Chinese Materia Medica, Jilin Agricultural Science and Technology University, Jilin, China; 7 The 3rd Affiliated Hospital of Changchun University of Chinese Medicine, Changchun, China

**Keywords:** drug discovery, hepatoprotection, hepatotoxicity, liver disease, phytochemicals, zebrafish model

## Abstract

The search for novel therapeutics for prevalent liver diseases such as metabolic dysfunction-associated steatotic liver disease, alcohol-related liver disease, and drug-induced liver injury is constrained by the methodological gaps in conventional preclinical models, which struggle to balance physiological complexity with screening efficiency. This challenge is particularly acute for natural products, where elucidating multifaceted mechanisms and inherent toxicological risks is paramount for translation. The zebrafish (*Danio rerio*) model, with its unique attributes of optical transparency, genetic tractability, and high-throughput capability, has emerged as a transformative platform to address this bottleneck. This review synthesizes and critically evaluates the integral role of zebrafish in advancing natural product-based hepatology. We provide a systematic analysis of established protocols for modeling key liver pathologies—from diet-induced and ethanol-induced steatosis to chemical hepatotoxicity—and consolidate evidence on how these models have been leveraged to decipher protective mechanisms, including the regulation of lipid metabolism, oxidative stress, and inflammation. Crucially, we integrate the parallel and essential discourse on safety, highlighting how zebrafish models, especially transgenic lines, enable the real-time visualization and mechanistic interrogation of compound-induced hepatotoxicity. By confronting current limitations, such as interspecies metabolic differences and protocol variability, we outline a strategic roadmap for the field. This involves the integration of multi-omics, humanized genetics, and standardized approaches to enhance the predictive validity of zebrafish studies. Ultimately, this review articulates how the zebrafish serves as a unified *in vivo* system to accelerate the identification and mechanistic validation of plant-derived therapeutics while concurrently de-risking their development, thereby directly contributing to the pipeline for new treatment options in liver disease.

## 1 Introduction

The liver is indispensable for systemic homeostasis, performing essential functions in metabolism, detoxification, and synthesis (Pellicoro et al., 2014). Consequently, its dysfunction—manifesting in prevalent conditions like metabolic dysfunction-associated steatotic liver disease (MASLD), alcohol-related liver disease (ALD), and drug-induced liver injury (DILI)—poses a severe and growing global health challenge. A critical barrier to addressing this challenge is the stark lack of approved pharmacotherapies for many liver disorders, as exemplified by the limited options for MASLD (Zhang and Yang, 2021). This therapeutic deficit is partly rooted in the limitations of conventional preclinical models. While physiologically relevant, traditional rodent studies are often low-throughput and resource-intensive (Cosgrove et al., 2009), whereas simplified *in vitro* systems cannot replicate the organ’s complex cellular interplay and spatial metabolism (Scheidecker et al., 2020). This disparity creates a pressing need for translational models that can bridge the gap between scalable screening and physiological relevance to accelerate the discovery of novel treatments.

In this quest for new therapies, plant-derived phytochemicals represent a promising frontier due to their vast chemical diversity and multi-target therapeutic potential (Guo et al., 2024). However, their development is hampered by methodological complexities, as their intricate compositions necessitate platforms that can efficiently evaluate both efficacy and the inherent risk of hepatotoxicity—a dual assessment crucial for clinical translation. Therefore, advancing liver drug discovery from natural sources depends on developing more efficient and insightful preclinical evaluation strategies.

The zebrafish (*Danio rerio*) model has emerged as a powerful vertebrate system uniquely suited to overcome these hurdles and provide direct insights into liver disease mechanisms. Beyond its approximately 70% protein-coding gene homology with humans and a highly conserved hepatobiliary system (Howe et al., 2013), the zebrafish offers methodological advantages that directly serve translational hepatology research (Salmi et al., 2019; Meguro et al., 2013; Lin et al., 2017). Its larval optical transparency allows for real-time, non-invasive imaging of dynamic pathological processes such as steatosis and inflammation. Furthermore, its small size and rapid development enable high-throughput studies that are impractical in rodents, aligning with ethical research principles. Crucially, these features do not merely make zebrafish a screening filter; they establish it as a dynamic platform for elucidating disease pathophysiology and mechanism of action.

Although zebrafish liver disease models are widely employed, a dedicated methodological synthesis evaluating their application specifically for phytochemical research—from model establishment to mechanistic validation—is lacking. This review aims to address this gap by providing a comprehensive critical framework. We will evaluate protocols for establishing key zebrafish liver injury models (MAFLD, ALD, DILI) and synthesize advances in associated biomarkers and mechanistic endpoints for testing phytochemicals. A central focus will be on integrating efficacy and safety assessments to distinguish genuine hepatoprotection from toxicity. By consolidating these strategic methodologies, this review aims to highlight how the zebrafish model can accelerate the translation of phytochemicals into credible therapeutic candidates, thereby contributing to the broader goal of understanding liver diseases and developing new treatment options.

## 2 Zebrafish models for uncovering therapeutic mechanisms of phytochemicals in metabolic dysfunction-associated steatotic liver disease

Metabolic dysfunction-associated steatotic liver disease (MASLD), formerly termed non-alcoholic fatty liver disease (NAFLD), has emerged as a prevalent global health burden, affecting approximately 30% of the adult population (Rinella and Sookoian, 2024). MASLD encompasses a spectrum of chronic liver conditions defined by excessive hepatic lipid accumulation in the absence of significant alcohol consumption, posing a major global health challenge (Guo et al., 2022; Shan et al., 2024; Mujica and den Hoed, 2023). Its pathogenesis, widely described by the “multiple-hit” theory, involves a complex interplay of metabolic dysregulation, insulin resistance, and inflammation, with gut microbiota dysbiosis also recognized as a critical contributor that can exacerbate disease progression through mechanisms like endotoxin translocation (Xue et al., 2017). The zebrafish has emerged as a particularly valuable model for deciphering this complexity and screening therapeutic candidates, as it allows for the rapid establishment of pathology through various dietary and chemical means that recapitulate key features of human MASLD.

Commonly employed dietary induction methods include high-cholesterol (HCD), high-fat (HFD), high-fructose (HFrD), and overfeeding (OF) protocols, each modeling distinct metabolic drivers of steatosis. For instance, the HCD model effectively induces hepatic cholesterol deposition and inflammation, making it suitable for studying the progression to steatohepatitis (NASH) and fibrosis (Ma et al., 2019). The HFD model mimics steatosis resulting from excessive fat intake (Li et al., 2024), while the HFrD model recapitulates impairments from high-sugar diets (Sapp et al., 2014). The OF model simulates the caloric surplus and metabolic imbalance central to metabolic syndrome (Li X. et al., 2023). These models are most frequently initiated in larvae, primarily due to their optical transparency, which permits non-invasive real-time imaging of pathological processes, their small size enabling high-throughput screening, and their rapid developmental timeline. However, adult zebrafish models are also valuable, particularly for studying chronic disease progression, fibrotic changes, and long-term dietary interventions, offering complementary insights that are not feasible in larval stages. Alternatively, chemical induction with agents like thioacetamide (TAA) can establish a MASLD-like injury phenotype encompassing inflammation, fibrosis, and metabolic dysfunction, providing another robust platform for mechanistic and therapeutic discovery (Amali et al., 2006; Salguero Palacios et al., 2008).

The utility of these models is clearly demonstrated in the evaluation of plant-derived phytochemicals (Table 1). Research across different models has been instrumental in elucidating a wide array of therapeutic mechanisms. In HCD-induced models, natural products are often shown to modulate cholesterol and lipid metabolism while counteracting inflammation and oxidative stress. For example, extracts from *Smilax glabra* Roxb. and *Cichorium glandulosum* have demonstrated comprehensive benefits by downregulating lipogenic genes and pro-inflammatory cytokines while enhancing antioxidant defenses and lipid catabolism (Li et al., 2018; Xiong et al., 2025). Furthermore, purified flavonoids and phenolics, such as kaempferol-3-O-glucuronide, puerarin, and esculetin, have been shown to alleviate metabolic disorders and activate protective pathways like Nrf2/Keap1 and PI3K/AKT, highlighting the model’s strength in delineating multi-target mechanisms (Ahmad et al., 2019; An et al., 2021; Deng et al., 2021; Fang et al., 2024; Haridevamuthu et al., 2023; Ma et al., 2023).

**TABLE 1:** Natural products screened for MAFLD therapy in zebrafish models.

Extracts/Compounds	Types of models	Details of models	Phase of action	Changes in indicators	References
Ethanol extracts of *Smilax glabra Roxb*. (SGR)	High-cholesterol-diet-(HCD) induced zebrafish modeling	Wild type AB zebrafish larve at 8dpf; Feeding with 5% HCD for 13 days	Improve lipid metabolism, anti-oxidative Stress, modulated intestinal microbiota composition	**↓:** TC, TG, LDLC, MDA, fasn, hmgcr, dgat2, IL-6, tnf-α, keap1 **↑:** TAOC, SOD, cyp7a1, cpt1a, cat, sod1, gstm3, nrf2	Xiong et al. (2025)
*Cichorium glandulosum Boiss. et Huet*	High-cholesterol-diet-(HCD) induced zebrafish modeling	Wild type AB zebrafish larve at 6dpf; Feeding with 5% HCD for 7 days	Inhibits lipogenesis	**↓:** TC, TG, srebf-1, fas **↑:** pparab	Li et al. (2018)
Kaempferol-3-O-glucuronide	High-cholesterol diet (HCD) induced zebrafish modeling	Wild-type AB-line and Tg (*mpx*: EGFP) zebrafish larve at 6dpf, 5% cholesterol-containing food	Improve lipid metabolism; Suppressing oxidative stress; alleviating inflammation;	**↓:** TG, TC, MDA, ROS, Nrf2/Keap1, il-1β, tnf-α and il-6 **↑:** GSH	Deng et al. (2021)
Puerarin	High-Cholesterol-diet-(HCD) induced zebrafish modeling	Wild type AB and Tg (zlyz-EGFP) zebrafish larve at 5 dpf Zebrafish larvae at 5 dpf were fed 10% HCD for 8 days	Regulation of macrophage polarization and autophagy Reduces hepatic lipid accumulation, inflammation, and fibrosis	**↓:**TG, TC, ALT, AST, IL-6, IL-1β, p-mTOR, p-STAT3, HIF-1α **↑:**IL-10, p-PI3K, p-AKT, p-AMPK, p-ULK1	Fang et al. (2024)
P- Hydroxybenzyl alcohol	High-cholesterol-diet-(HCD) induced zebrafish modeling	Wild type AB zebrafish larve at 6dpf Fed with 5% HCD (20 mg/tank per day) for 7 days	Performing lipid regulation and improve oxidative stress	**↓:** TC, TG, ROS, MDA **↑:** SOD, HO-1	An et al. (2021)
Gastrodin	High-cholesterol-diet-(HCD) induced zebrafish modeling	Wild type AB zebrafish larve at 6dpf; Feeding with 5% HCD for 7 days	Improving lipid metabolism, Anti-oxidative stress	**↓:**TG, TC, ROS, MDA	Ahmad et al. (2019)
Esculetin	High-cholesterol-diet-(HCD) induced zebrafish modeling	Wild type AB zebrafish at 6dpf 4% high cholesterol diets treatment with 13 days	Improving lipid metabolism, Anti-oxidative stress, Anti-inflammatory effect	**↓:** TC, TG, ROS MDA, srebp1c **↑:** GSH, PPARγ, HO-1	Ma et al. (2023)
Piperlongumine	High-cholesterol- diet (HCD) induced zebrafish modeling	Wild type AB zebrafish at 6dpf; 4% High Cholesterol Diets Treatment with 5 days	Improving lipid metabolism, Anti-oxidative stress, Anti-inflammatory effect	**↓:** TC, TG, ROS, MDA, srebp1, fasn, cebpa, tnf-α, il1-β, il-6 **↑:** SOD, CAT, GSH, pparab, pparg	Haridevamuthu et al. (2023)
Dimeric guaianolide sesquiterpenoids	High-fat diet (HFD)-induced zebrafish model	Wild type AB zebrafish larve at 8dpf; Feeding a high-fat diet with egg yolk as the main source	Activation of SIRT1 Pathway; Inhibition of lipogenesis; Promotion of fatty acid oxidation; Inhibition of ferroptosis	**↓:** TG, ROS, MDA, ALT, AST, NEFA, T-CHO **↑:** GSH, SOD	Liu et al. (2025)
Fucoidan	High-fat diet (HFD)-induced zebrafish modeling	CD36 transgenic zebrafish larve at 5dpf Feeding a high-fat diet for 10 days	Preventing cell proliferation, lipid metabolism, and inflammation	**↓:** srebf1, hmgcs1, acox3, atf6, il6, ccne1	Wu et al. (2020)
B-type procyanidins	High-fat diet (HFD)-induced zebrafish modeling	Wild type AB zebrafish larve at 20 dpf Feeding a high-fat diet for 14 days	Reduce lipid accumulation and oxidative stress levels, enhance fatty acid oxidation, and improve mitochondrial function	**↓:** srebp1c, FAS, ACC, SCD1, PPARγ, TG, TC, p-mTOR, MDA, ROS **↑:** p-AMPKα, p-JNK, PPARα, Nrf2, HO-1, GSH, SOD	Tie et al. (2025)
Chlorogenic acid and its isomers	High-fat diet (HFD)-induced zebrafish modeling	Zebrafish were fed a high-fat diet containing 2% cholesterol starting at 20 dpf for 14 days	Regulation of lipid metabolism; Reduction of oxidative stress; Improvement of blood lipid profile	**↓:** pmTOR, srebp1c, FAS, ACC, SCD-1, PPARγ, TG, TC, pmTOR MDA, ROS **↑:** p-AMPKα, p-JNK, PPARα, Nrf2, HO-1, GSH, SOD	Tie et al. (2024)
β-Sitosterol	High-fat diet (HFD)-induced zebrafish modeling	5dpf zebrafish larvae were fed a high-fat diet for 5 days and immersed in a 3% glucose solution Adult zebrafish were fed a high-fat diet for 1 month	Inhibits fat production; Regulate lipid metabolism; Improve sugar metabolism; Anti-inflammatory and antioxidant effect	**↓:** TG, TC, Glucose, PPAR-γ, RXR-α	Zhang et al. (2024b)
Dried tangerine peel polysaccharide	High-cholesterol and high-fructose (HCF) diet-induced masld model	5 dpf zebrafish larve were fed an HCF diet (containing 10% cholesterol and 4% fructose) for 3 days Adult zebrafish were fed an HCF diet for 5 weeks	Improving lipid metabolism; Antioxidant effects; Anti-inflammatory properties; Enhancing mitochondrial function	**↓:** TG, TC, TNF-α, IL-6, MDA **↑:** SOD, GSH-Px, ATP, IL-10	Wang et al. (2024)
Eriocitrin	Over feeding (OF) Induced zebrafish modeling	Wild type AB adult zebrafish at 3.5 mpf Fed three times per day with Artemia	Ameliorates diet-induced hepatic steatosis with activation of mitochondrial biogenesis	**↓:** TC, TG, mtTFA, NRF1, COX4, ATP synthase	Hiramitsu et al. (2014)
Resveratrol	Over feeding (OF) Induced zebrafish modeling	Wild type AB adult zebrafish; fed with freshly hatched live artemia	Promoting lipid decomposition; enhanceing autophagy and inhibiting adipogenesis	**↓:** TG, PPARγ, Caveolin-1, pAMPKα/AMPKα **↑:** Sirt1, LC3-II	Ran et al. (2017)
Caffeine	Over feeding (OF) Induced zebrafish modeling	Wild type AB zebrafish larve at 5dpf	Inhibition of steatosis and reduction of lipid accumulation	**↓:** TC, SREBP1, ACC1, CD36, UCP2, PERK, IRE1, ATF6, BIP, CHOP, BIP, IL-1	Zheng et al. (2015)
Limonin	Thioacetamide (TAA) induced zebrafish modeling	Wild-type AB-line,Tg (lfabp10α: eGFP) and Tg (mpeg1: dsred) zebrafish 0.4 mg/mL thioacetamide treatment for 72 h	Improve lipid metabolism; Suppressing oxidative stress; Alleviating inflammation	**↓:** fasn, srebp1, IL-6, IL-1β and TNF-α, NRF2/HO-1	Li et al. (2021b)
Crocetin	Thioacetamide (TAA) induced zebrafish modeling	Wild type AB zebrafish larve 1,000 μM TAA treatment for 72 h	Restoring mitochondrial morphology Reverses the accumulation of lipid droplets	**↓:** ATP, cleaved caspase-3, Bax **↑:** Bcl-2, COX IV	Xu et al. (2022)
New sesquiterpenes and viridin derivatives	Thioacetamide (TAA) induced zebrafish modeling	Wild type AB and Tg(Ifabp10acGFP) Zebrafish larvae at 3 dpf were treated with 0.4 mg/mL TAA for 72 h	Reduce lipid accumulation Activation of the PINK1/Parkin mitophagy pathway	**↓:** ALT, AST, LDL-C, TG **↑:** HDL-C, ATP	Zhang et al. (2024a)
Wedelolactone and demethylwedelolactone	Thioacetamide (TAA) induced zebrafish modeling	Tg(Ifabp10: DsRed) zebrafish larvae Zebrafish larvae (72 hpf) were exposed to 10 mM TAA until 120 hpf	Inhibition of steroid biosynthesis Regulates fatty acid metabolism Improve energy metabolism	**↓:** AST, TG, Ebp, dgat2 **↑:** pnpla3	Chen et al. (2024)
Lycii Fructus Polysaccharide	Thioacetamide (TAA) induced zebrafish modeling	Wild type AB zebrafish embryos at 10 hpf and Tg (Ifabp10a: eGFP) transgenic lines 7 mM TAA treatment for 72 h	Suppressing oxidative stress, Alleviate abnormal cell apoptosis	**↓:** ROS, NO, MDA, ALT, AST, Bax, Caspase-3, c-myc **↑:** SOD, CAT, GSH-Px, GSH, T-AOC, Bcl-2	Zhang et al. (2021)
Ellagic acid	Iodoacetamide (IAA) induced zebrafish modeling	Wild type AB adult zebrafish Fishes were injected twice (1st and 6th day) with 300 mg/kg bodyweight IAA in saline	Promoting lipid metabolism and inhibiting fat accumulation	**↓:** MDA, ALT, AST, TC, TG **↑:** GSH	Venkatasubramanian et al. (2021)

These abstract uses wild-type and transgenic zebrafish as experimental models to provide information on the regulatory effects of natural product extracts or monomeric components on non-alcoholic fatty liver disease. “↑” or “↓” indicates the regulatory effects of natural product extracts and their monomeric components on different indicators. “↑” indicates increase or upregulation; “↓” indicates decrease or downregulation.

Conversely, studies utilizing HFD-induced models have primarily revealed how compounds promote fatty acid oxidation and improve mitochondrial function. Dimeric guaianolide sesquiterpenoids and fucoidan, for instance, have been found to enhance lipid breakdown and mitigate inflammation (Liu et al., 2025; Wu et al., 2020), while procyanidins and chlorogenic acids activate signaling pathways such as Nrf2/HO-1 to boost antioxidant capacity and metabolic homeostasis (Tie et al., 2024; Tie et al., 2025). Similarly, the natural phytosterol β-sitosterol exerts hypolipidemic effects by modulating pathways related to adipogenesis and lipid catabolism (Zhang P. et al., 2024). Other dietary models yield further insights; for example, in a high-cholesterol and high-fructose diet model, polysaccharides like those from dried tangerine peel showed hepatoprotection through antioxidative and anti-inflammatory actions (Wang et al., 2024).

Investigations using other modeling approaches continue to expand our understanding. In an overfeeding (OF) model, compounds like eriocitrin and resveratrol were found to protect the liver by enhancing mitochondrial biogenesis and activating autophagy pathways, respectively (Hiramitsu et al., 2014; Ran et al., 2017). In chemical models, such as those induced by TAA or iodoacetamide, a diverse set of compounds including limonin, crocetin, wedelolactone, polysaccharides, and ellagic acid have demonstrated significant hepatoprotection through anti-inflammatory, antioxidative, and anti-apoptotic actions, often involving the regulation of mitochondrial quality control and key metabolic genes (Chen et al., 2024; Li et al., 2021b; Venkatasubramanian et al., 2021; Xu et al., 2022; Zhang and Yang, 2021; Zhang H. et al., 2024). Early studies also support the utility of models like caffeine in an OF context for studying lipogenic gene regulation (Zheng et al., 2015).

In conclusion, the collective research across these varied zebrafish MASLD models underscores their exceptional relevance and sensitivity for disease modeling. They provide a versatile *in vivo* platform that does more than just screen for bioactivity; it actively helps deconstruct the multifaceted mechanisms of liver pathology. The consistent findings—that phytochemicals like flavonoids, phenolic acids, and polysaccharides can mitigate MASLD by regulating lipid metabolism, oxidative stress, and inflammation—validate the zebrafish as a powerful tool for bridging the gap between the discovery of natural compounds and the development of novel, mechanism-informed treatment strategies for liver disease.

## 3 Zebrafish models for uncovering therapeutic mechanisms of phytochemicals in alcohol-related liver disease

Alcohol-related liver disease (ALD), a major healthcare burden stemming from chronic excessive alcohol consumption, progresses through a complex pathogenesis involving metabolic disruption, oxidative stress, and inflammation. The hepatic metabolism of ethanol *via* enzymes like ADH and CYP-2E1 generates acetaldehyde and reactive oxygen species (ROS). When ROS production overwhelms endogenous antioxidant systems, it induces significant oxidative stress and steatosis (Ceni et al., 2014; Tao et al., 2021). Concurrently, chronic ethanol intake dysregulates key lipid metabolism genes, promoting fatty acid synthesis and suppressing β-oxidation, which leads to pathological triglyceride accumulation in hepatocytes (You and Arteel, 2019). This metabolic insult is compounded by ROS-induced endoplasmic reticulum stress, marked by the upregulation of UPR components like CHOP and GRP78, which further promotes hepatocyte apoptosis and inflammatory activation (Zhang et al., 2022). The histopathological hallmarks of ALD include steatosis, hepatocyte ballooning, and neutrophil infiltration (Stickel et al., 2017), mirrored biochemically by elevated serum transaminases, dyslipidemia, diminished antioxidant enzyme activities, and increased pro-inflammatory cytokines (Li et al., 2025). An additional critical layer of pathophysiology involves alcohol-induced gut barrier dysfunction and microbiota dysbiosis, which promotes endotoxin translocation to the liver, thereby exacerbating immune-mediated inflammation and driving progression from steatosis to hepatitis and fibrosis (Wang et al., 2010).

The zebrafish model has proven highly effective for deconstructing this multifaceted pathology and for screening therapeutic interventions (Table 2). Typically utilizing 3–5 days post-fertilization (dpf) larvae of wild-type AB or liver-specific transgenic lines, researchers establish acute alcoholic liver injury models primarily through direct ethanol exposure. A common, though not fully standardized, protocol involves exposing larvae to 1%–2% (v/v) ethanol for 24–48 h, which rapidly induces hepatic lipid accumulation, oxidative stress, and inflammatory responses (Tsedensodnom et al., 2013). A systematic evaluation of time- and dose-dependent effects has been provided by Shihana et al., highlighting the importance of protocol optimization for reproducible pharmacological screening (Shihana et al., 2023). However, a key methodological consideration for reproducible pharmacological screening is the need to optimize and standardize ethanol concentration and exposure duration to robustly induce steatosis while minimizing systemic toxicity and mortality. The model’s strength lies in its capacity to move beyond simple phenotypic screening to provide direct, real-time insights into the mechanistic actions of potential therapeutics, as evidenced by research on diverse phytochemicals.

**TABLE 2:** Evaluation of natural products against alcohol-related liver disease in ethanol-induced zebrafish models.

Extracts/Compounds	Details of models	Phase of action	Changes in indicators	Ref(s)
Fermented ginseng	Wild type AB zebrafish larve at 96hpf 30% ethanol fed 0.1 mL treatment for 32 h	Enhancing the GSH-PX and SOD activities and reduced the MDA production in the liver	**↑**: GSH-PX, SOD **↓**: MDA	Xing-Yu et al. (2022)
*Penthorum chinense Pursh*	Tg (fabp10: EGFP) zebrafish larvae at 3dpf 350 mmol/L ethanol treatment for 32 h	Inhibiting the synthesis of fatty acids and oxidative Stress Increasing in autophagy	**↑**: SIRT1, PPARα, GSH, CAT, SOD, Keap1/Nrf2 ↓: PPARγ, CPT1, ROS, MDA, PI3K/Akt/mTOR	Zhao et al. (2020)
Essential oil from *Artemisia argyi*	Wild type AB adult zebrafish	Relieving ethanol-caused histopathological damage of livers by modulating the composition of gut microbiota Attenuating the damage to intestinal tissue structure	**↑**: CAT, SOD, GSH, IL-10, IFN-γ **↓**: MDA.IL-1β, IL-6, PPAR-γ, NF-κB, TNF-α	Chen et al. (2023)
*Puerariae Lobatae radix* flavonoids puerarin	Wild type AB zebrafish larvae at 5dpf 2% Ethanol treatment for 32 h	Reducing hepatic steatosis Improving alcohol metabolism and lipid metabolism Reducing endoplasmic reticulum stress and DNA damage	**↓**: CYP2y3, CYP3a65, ADH8a, ADH8b, HMGCRB, FASN, CHOP, EDEM1, GADD45αa and ATF6 IL-1β, TNF-α, ACC1, TC, TG **↑**: AMPKα	Liu et al. (2021)
Hesperidin	wildtype AB and Tg (fabp10: eGFP) zebrafish larvae at 4dpf 350 mM ethanol treatment for 32 h	Reducing hepatic steatosis Improving alcohol metabolism and lipid metabolism Reducing endoplasmic reticulum stress and DNA damage	**↓**: Alcohol and lipid metabolism related genes (cyp2y3, cyp3a65, hmgcra, hmgcrb, fasn, fads2) **↓**: Endoplasmic reticulum stress and DNA damage related genes (chop, gadd45*α*a, edem1)	Zhou et al. (2017)
Narirutin	Wild type AB larvae between 96 and 98 hpf 1% ethanol treatment for 48 h	Preventing lipid formation, protecting the antioxidant system; suppressing endoplasmic reticulum stress;	**↓**: tnf-α, nf-κB, il-1β, casp3a, dffa, mapk14, col1a1a, cyp2p6, cyp2y3, nox1, nox2, nrf2, sod2, gsr, gpx1a, prdx4, txnl4, srebp1, srebp2, cebpa, acc1, fasn, daat2, cpt1a, lpl, ldlr, atf4, atf6, ire1, xbp1-u, xbp1-s, bip, dnajc3, grp94, ddit3, edem1, gadd45a, ALT, AST, MDA, TG	Park et al. (2023)
Quercetin	Tg (fabp10: EGFP) zebrafish larvae at 5dpf 350 mmol/L ethanol treatment for 32 h	Inhibiting acute ethanol-induced hepatic steatosis and hepatic oxidative stress	**↓**: ALT, AST, γ-GT, TG, TC, ROS, MDA, GSH, CAT, SOD, P2X7, Keap1 **↑**: PI3K, Nrf2, Akt, CAT1, GPX8, NQO1	Zhao et al. (2021)
Polydatin	Wildtype AB and Tg (lfabp10-α: eGFP) zebrafish larvae at 4dpf 350 mM ethanol (2% EtOH) treatment for 32 h at 28.5 C	Attenuating hepatic fat accumulation Ameliorating lipid and ethanol metabolism Reducing oxidative stress and DNA damage	**↓**: CYP2Y3, CYP3A65, HMGCRa, HMGCRb FASN, chop, gadd45*α*a	Lai et al. (2018)
Fucoidan	Wild type AB zebrafish larvae at 96hpf 1.5% Ethanol treatment for 32 h	Decreasing oxidative stress and lipid peroxidation	**↓**: MDA, ROS, p53 **↑**: GSH	Dai et al. (2020)
Rutin	Tg(mito:EGFP) zebrafish larvae at 4dpf 2% ethanol treatment for 24 h	Inhibiting acute ethanol-induced hepatic steatosis Restoring the mitochondrial networks to normal status	**↓**: c/ebpα, pparγ, DRP1 **↑**: MFN2	Choi et al. (2021b)
Ginsenoside Rb1	Wild type AB zebrafish and Tg (lfabp10α: EGFP) and Tg (MPO: EGFP) larvae at 4dpf 350 mM ethanol treatment for 32 h	Inhibiting Steatosis, oxidative stress and inflammation	**↓**: NF-κB, TNF-α, ROS, MDA	Lai et al. (2021)

These abstract uses wild-type and transgenic zebrafish as experimental models to provide information on the regulatory effects of natural product extracts or monomeric components on alcoholic fatty liver disease. “↑” or “↓” indicates the regulatory effects of natural product extracts and their monomeric components on different indicators. “↑” indicates increase or upregulation; “↓” indicates decrease or downregulation.

Studies leveraging this model have shown that plant extracts exert hepatoprotective effects through multi-target synergistic actions. For instance, fermented ginseng enhances the hepatic antioxidant capacity by increasing activities of enzymes like GSH-Px and SOD (Xing-Yu et al., 2022). The extract of Penthorum chinense Pursh mitigates ethanol-induced lipid accumulation and oxidative damage by activating the SIRT1/PPARα and Keap1/Nrf2 pathways to promote fatty acid β-oxidation, while concurrently inhibiting the PI3K/Akt/mTOR-mediated lipid synthesis (Zhao et al., 2020). Furthermore, the essential oil of Artemisia argyi demonstrates a holistic protective role by not only attenuating liver tissue injury but also modulating gut microbiota and restoring intestinal barrier function, which leads to a reduction in pro-inflammatory mediators and an increase in anti-inflammatory factors (Chen et al., 2023).

Research on purified bioactive compounds has been particularly revealing of specific molecular mechanisms. Flavonoids, a prominent class, show consistent efficacy. Puerarin and hesperidin, for example, enhance ethanol metabolism and suppress lipogenesis by modulating alcohol-metabolizing and lipogenic genes, while also suppressing endoplasmic reticulum stress and DNA damage pathways to mitigate apoptosis (Liu et al., 2021; Zhou et al., 2017). Narirutin exhibits notable anti-inflammatory and antioxidant properties by downregulating pro-apoptotic and pro-inflammatory genes and upregulating Nrf2-dependent antioxidant defenses (Park et al., 2023). Similarly, quercetin attenuates oxidative injury and lipid accumulation *via* activation of the PI3K/Nrf2/Akt signaling pathway (Zhao et al., 2021). Other polyphenols, such as polydatin, alleviate oxidative and DNA damage by downregulating key ethanol-metabolizing and lipogenic enzymes (Lai et al., 2018). Polysaccharides like fucoidan from brown algae strengthen cellular antioxidant capacity and maintain redox homeostasis to counteract ethanol-induced damage (Dai et al., 2020). Additional monomeric compounds, including rutin and ginsenoside Rb1, have been shown to alleviate steatosis and inflammation through distinct pathways, such as inhibiting lipidogenesis and restoring mitochondrial integrity or blocking *NF-κB* signaling (Choi Y. et al., 2021; Lai et al., 2021; Zhao et al., 2021).

In summary, the ethanol-induced zebrafish model has established itself as a vital platform not merely for screening but for mechanistically dissecting ALD. It enables researchers to visualize how phytochemicals—from complex extracts to pure monomers—intervene at multiple levels. These interventions include modulating alcohol metabolism, reprogramming lipid homeostasis, activating endogenous antioxidant and autophagy pathways, and mitigating endoplasmic reticulum stress and inflammation. The consistent findings across these studies validate the model’s reliability and its unique utility in bridging the gap between the observed hepatoprotection and the underlying molecular pathogenesis, thereby providing a solid experimental foundation for the development of mechanism-informed natural product-based therapies for ALD.

Importantly, recent clinical perspectives have highlighted a significant overlap between ALD and metabolic liver disease, termed Met-ALD, which describes patients with both alcohol consumption and underlying metabolic risk factors (Bandyopadhyay et al., 2025). The zebrafish model, with its capacity to simultaneously manipulate dietary intake and ethanol exposure, is uniquely positioned to model this hybrid pathophysiology. Future studies incorporating combined high-fat or high-fructose diets with chronic ethanol exposure could provide critical insights into the synergistic mechanisms driving Met-ALD progression and facilitate the discovery of phytochemicals effective against this complex disease spectrum.

## 4 Zebrafish models for mechanistic insights into drug-induced liver injury and phytochemical protection

Drug-induced liver injury (DILI) represents a significant clinical challenge, arising from the direct or indirect hepatocellular damage caused by pharmaceuticals or their metabolites (Andrade et al., 2019). Its multifactorial pathogenesis encompasses metabolic activation, oxidative stress, inflammatory responses, and programmed cell death. Most xenobiotics undergo hepatic biotransformation *via* the CYP450 system, generating reactive intermediates that may form protein adducts or stimulate excessive production of reactive oxygen species (ROS), triggering downstream events like lipid peroxidation, mitochondrial dysfunction, and endoplasmic reticulum stress that collectively led to hepatocellular damage (Jaeschke et al., 2012; Russmann et al., 2009). Histopathological hallmarks include hepatic swelling, vacuolar degeneration, and necrosis, while clinical markers feature elevated serum transaminases (ALT, AST) and bilirubin, alongside reduced albumin, indicating compromised liver function (Andrade et al., 2019). Oxidative stress-induced mitochondrial impairment and glutathione (GSH) depletion are considered pivotal drivers of hepatocyte apoptosis and necrosis (Hinson et al., 2009), processes further exacerbated by the infiltration of inflammatory cells and the overproduction of pro-inflammatory cytokines such as *TNF-*α, *IL-1*β, *IL-6*, and *IL-8*, alongside anti-inflammatory mediators like *IL-10* (Hinson et al., 2009).

The zebrafish model has emerged as a powerful system for deconstructing this complexity (Table 3), owing to its high evolutionary conservation in liver morphology and function, including a well-characterized suite of CYP450 enzymes and a drug metabolism system that parallels human pathways (Goldstone et al., 2010). By 3 days post-fertilization, the zebrafish liver is functionally mature and responsive to drug stimuli, enabling robust *in vivo* DILI studies in larvae. Researchers employ various chemical inductors, such as thioacetamide (TAA), acetaminophen (APAP), isoniazid (INH), and iodoacetamide (IAA), each reliably inducing a spectrum of injury features—including lipid deposition, oxidative stress, inflammation, and apoptosis—while allowing for integrated evaluation through live imaging, biochemical assays, and molecular analyses. This system, therefore, provides more than a screening platform; it serves as a dynamic tool for uncovering the mechanistic interplay between specific toxic insults, the resulting pathophysiology, and potential interventions.

**TABLE 3:** Hepatoprotective natural products in chemically-induced liver injury zebrafish models.

Extracts/Compounds	Types of models	Details of models	Phase of action	Changes in indicators	Ref(s)
*Salvia plebeia* R. Br.	Thioacetamide (TAA) induced zebrafish modeling	Wild-type AB-line zebrafish embryos at 6hpf; 5mg/mLThioacetamide treatment for 72 h	Anti-oxidative stress; Anti-inflammatory effect; Reduce lipid deposition and liver fibrosis	**↓:** ROS, CAT, SOD, TNF-α, IL-β, NO, Cytochrome p450, ApoE, PPAR-γ, Bcl2, NF-κB **↑:** HO-1, Ac H3	Xiong et al. (2019)
*Curcuma phaeocaulis* Val.	Thioacetamide (TAA) induced zebrafish modeling	Wild type AB zebrafish larve at 72hpf 8 mmol/L TAA treatment for 72 h	Ameliorate TAA-induced zebrafish liver injury through TLR4/MyD88/NF-κB signaling pathway.	**↓:** ROS, IL-1β, IL-6, TNF-α, NF-κB, TLR4, MYD88, NF-κB-p65 **↑:** ALT, AST, SOD	Gao et al. (2024b)
Baicalein	Thioacetamide (TAA) induced zebrafish modeling	Wild type AB zebrafish embryos at 10 hpf and the Tg (fabp10a: DsRed) and Tg (fabp10a: eGFP) transgenic lines 0.4 mg/mL thioacetamide treatment for 48 h	Improve inflammatory response; reduce oxidative stress levels; regulates MAPK signaling pathway; increase p38, ERK1/2 and PPARa transcriptional activity	**↓:** TC, MDA, NO, IL-8, IFN-γ, Bax, p53 **↑:** TG, SOD, CAT, G6PDH, AchE, IL-1β, TNF-α, Bcl2, p38 MAPK, ERK2, ERK1, PPARα	Zhang et al. (2020)
Chlorogenic acid	Thioacetamide (TAA) induced zebrafish modeling	Tg(fabp10a: DsRed) zebrafish embryos at 10hpf; 0.4 mg/mL thioacetamide treatment for 38 h	Inhibit oxidative stress and cell apoptosis; promote cell proliferation; inhibit the expression of Wnt signaling pathway gene Dkk1; promote the expression of Lef1 and Wnt2bb	**↓:** MDA, ROS, NO, Bax, P53, Dkk1, Dkk2 **↑:** AchE, SOD, CAT, G6PDH, Bcl2, Lef1, Wnt2bb	Liu et al. (2022)
Livogrit	Thioacetamide (TAA) induced zebrafish modeling	Wild type AB adult zebrafish 45 μg/L TAA treatment for 14 days	Restore liver metabolism and excretion function, directly reduce liver cell damage	**↓:** AST, Bilirubin, Creatinine, INR, platelet clotting time, Liver dysfunction index **↑:** Serum Albumin, Sodium	Balkrishna et al. (2022)
Chebulinic acid	Acetaminophen (APAP) induced zebrafish modeling	Tg(fabp10a: dsRed; ela3l: EGFP) transgenic zebrafish embryos at 10 hpf 10 mM APAP treatment for 72 h	Alleviate oxidative stress; activate MAPK signaling pathway	**↓:** MDA, ROS, LDH **↑:** SOD, NQO1, Nrf2, pERK, pJNK, PP38	Feng et al. (2021)
Forsythiaside A	Acetaminophen (APAP) induced larval zebrafish modeling	Tg (L-FABP: EGFP) transgenic zebrafish larvae at 48hpf; 8 mM acetaminophen treatment for 48 h	Alleviate APAP-induced liver injury by regulating PI3K/AKT-mediated apoptosis	**↓:** ALT, AST, tnf, ask1, jnk, ap-1, caspase 3, caspase 8, caspase 9, akt, bax, pi3k, mmp9, mmp2 **↑:** GSH, bcl-2	Gong et al. (2021)
Hydroxysafflor yellow A and C (HSYA and HSYC)	Acetaminophen (APAP) induced larval zebrafish modeling	Wild type AB zebrafish and Tg(fabp10a: dsRed; ela3l: EGFP) transgenic zebrafish at 3 dpf 10 mM APAP treatment for 36 h	Improve local microcirculation of the liver Speeds up the elimination of acetaminophen	**↓:** ALT, AST, TNF-α, NF-κB p65, HIF-1α, iNOS, α-SMA **↑:** Liver size, Heart rate, Blood flow	Wang et al. (2021)
*Hedyotis diffusa* Willd.	Isoniazid (INH) induced zebrafish modeling	(L-FABP: EGFP) transgenic zebrafish larve at 3 dpf; 4 mM INH treatment for 72 h	Improve hepatocyte cytoplasm loosening, nuclear atrophy, vacuolization and inflammatory cell aggregation Inhibit oxidative stress	↓: ALT, AST ↑: GSH, SOD	Wang et al. (2023)
Isoliquiritigenin	Emodin (EMO) induced zebrafish modeling	Tg (L-FABP: EGFP) transgenic zebrafish embryos at 3dpf 1.8 μmol/L EMO treatment for 72 h	Attenuates the EMO-induced Nrf2 nuclear transfer, Alleviates the EMO-induced mitochondrial injury, Attenuates the EMO-induced Nrf2 nuclear transfer, dmitochondrial injury and oxidative stress	**↓:** Bax, MDA **↑:** Bcl-2, GSH, SOD, p-ERK, p-p38, Nrf2, Keap1, GST, NQO1, HO-1, MRP2, MRP4, BSEP	Ni et al. (2022)

These abstract uses wild-type and transgenic zebrafish as experimental models to provide information on the regulatory effects of natural product extracts or monomeric components on drug-induced liver injury. “↑” or “↓” indicates the regulatory effects of natural product extracts and their monomeric components on different indicators. “↑” indicates increase or upregulation; “↓” indicates decrease or downregulation.

Utilizing these models, studies on phytochemicals have systematically revealed multi-target protective mechanisms, highlighting their promise for DILI intervention (Zhou et al., 2021). These natural products generally exhibit convergent activities aimed at restoring redox balance, suppressing inflammation, inhibiting apoptosis, and promoting tissue repair. For instance, in the TAA-induced model, which often progresses to fibrosis, plant extracts like those from *Salvia plebeia* R. Br. and *Curcuma phaeocaulis* Val. have demonstrated potent regulatory effects. They significantly decrease markers of oxidative stress (ROS, NO) and inflammation (*TNF-*α, *IL-1*β), while modulating pathways involved in lipid metabolism (*PPAR-γ*) and antioxidant defense (*HO-1*, *Nrf2*), with the latter extract specifically shown to suppress the TLR4/MyD88/NF-κB signaling pathway (Gao T. et al., 2024; Xiong et al., 2019; Manuneedhi Cholan et al., 2022).

Research on purified compounds in TAA models further refines our understanding of these mechanisms. Flavonoids such as baicalein activate p38 MAPK and ERK1/2 pathways to upregulate PPAR-α expression, thereby enhancing antioxidant enzyme activities and modulating apoptosis-related proteins (Zhang et al., 2020). Phenolic compounds like chlorogenic acid exhibit combined antioxidant and anti-apoptotic effects, partly by modulating the Wnt pathway to facilitate tissue repair (Liu et al., 2022). A compound preparation like Livogrit has been shown to exert systemic hepatoprotection in adult zebrafish, restoring metabolic and secretory functions (Balkrishna et al., 2022). These findings illustrate how the model helps delineate compound-specific pathways within a common injury context.

In contrast, the APAP-induced model typically mimics acute hepatocellular necrosis driven by the toxic metabolite NAPQI, which depletes GSH and causes severe mitochondrial oxidative damage (Ramachandran and Jaeschke, 2019). Studies in this context highlight how natural products can precisely target this cascade. Chebulinic acid, for example, activates the Nrf2 and MAPK signaling pathways to upregulate endogenous antioxidants like SOD and NQO1 (Feng et al., 2021). Forsythiaside A exerts protection by intricately modulating apoptotic signaling, inhibiting the ASK/JNK/AP-1 axis while enhancing the pro-survival PI3K/Akt pathway (Gong et al., 2021). Similarly, compounds like Safflower yellow A and C aid recovery by improving hepatic microcirculation and suppressing the HIF-1α/iNOS axis, facilitating toxin excretion (Wang et al., 2021).

Other chemical models provide additional mechanistic insights. In the INH-induced model, associated with mitochondrial dysfunction and oxidative stress from metabolite-protein binding (Zhuang et al., 2022), extracts like that from Hedyotis diffusa Willd. show efficacy by alleviating histopathological damage and counteracting oxidative stress (Wang et al., 2023). Furthermore, research using models where anthraquinones like emodin (EMO) themselves induce injury has revealed how protective flavonoids such as isoliquiritigenin work by upregulating cellular detoxification and antioxidant defenses *via* the Nrf2/Keap1 pathway, showcasing the model’s utility in studying herb-herb interactions or intrinsic toxicity mitigation (Ni et al., 2022).

Therefore, the collective evidence from chemically induced zebrafish DILI models confirms more than just the efficacy of natural products; it validates the zebrafish as an indispensable platform for mechanistic discovery. These studies consistently demonstrate that phytochemicals—from complex extracts to single compounds—converge on core pathological processes: they enhance antioxidant systems, suppress inflammatory and apoptotic cascades, and promote repair. By enabling the dissection of pathway-specific regulatory differences across diverse toxic insults, the zebrafish model provides profound insights into both DILI pathophysiology and the multi-target therapeutic strategies offered by natural products, thereby contributing significantly to the field of hepatoprotective drug discovery and safety assessment.

## 5 The dual role of zebrafish models: uncovering therapeutic potential and evaluating hepatotoxic risk

While the preceding sections highlight the promising hepatoprotective effects of phytochemicals, a comprehensive assessment of any potential therapeutic agent must rigorously evaluate its safety profile. This dual focus on efficacy and toxicity is particularly crucial for natural products, as their complex composition and widespread use often foster a perception of inherent safety that may not align with pharmacological reality. Therefore, safety evaluation forms a critical foundation in early-stage pharmacological research on natural products (Jayasinghe and Jayawardena, 2019). Given that the liver is the primary site for the metabolism and clearance of most xenobiotics, assessing potential hepatotoxicity is essential for determining the feasibility and risk profile of any candidate compound destined for drug development (Almazroo et al., 2017; Frenzel and Teschke, 2016). In this context, the zebrafish model demonstrates its versatile utility by serving as an equally powerful platform for mechanistic toxicology, having evolved into an important branch of preclinical safety research (Modarresi Chahardehi et al., 2020).

Standard hepatotoxicity assessment in zebrafish integrates multidimensional indicators. In embryos or larvae, initial screening evaluates gross developmental endpoints such as mortality, malformation rates, and behavioral alterations. Concurrently, liver-specific analyses are performed; in wild-type larvae, whole-mount or histological staining (e.g., Oil Red O, H&E) enables the observation of hepatocyte morphology and lipid accumulation. The use of transgenic fluorescent lines, such as Tg (fabp10a: eGFP), significantly enhances sensitivity, allowing for real-time, non-invasive monitoring of hepatic integrity by quantifying fluorescence attenuation, morphological contraction, or changes in fluorescence intensity (Choe et al., 2021). Beyond phenotype, biochemical and molecular analyses provide mechanistic depth. Common serum biomarkers like ALT, AST, and total bile acids reflect hepatocellular membrane integrity and cholestatic injury, while oxidative stress parameters (ROS, MDA, SOD, CAT, GSH) and inflammatory cytokines (*il-1β*, *tnf-α*, *il-6*) help elucidate the cellular pathways driving damage (Bojarski et al., 2025; Marginean, 2025; Metri et al., 2025). Furthermore, high-throughput omics techniques—including genomics, proteomics, and metabolomics—combined with bioinformatics offer powerful tools for systematically investigating the molecular mechanisms underlying toxicity (Martinez-Sena et al., 2023).

Employing this integrated framework, zebrafish studies have revealed that although many plant-derived constituents are relatively safe (Table 4), certain extracts and purified monomers exhibit significant dose-dependent hepatotoxicity, warranting careful attention (Jităreanu et al., 2022). Research in larval models has identified several concerning agents. For instance, fractions from Radix Sophorae tonkinensis and the 70% ethanol extract of Polygonum multiflorum Thunb. induce liver darkening, reduced hepatic area, and toxicity primarily attributed to anthraquinone constituents (Li et al., 2020; Liu et al., 2017; Yang et al., 2018). Extracts of Phytolacca acinosa Roxb. and Euphorbia kansui elevate serum transaminases and induce hepatocyte vacuolation and apoptosis, often through caspase-3-dependent pathways (Cao et al., 2021; Zhao et al., 2019). Similarly, *Psoralea corylifolia* L. extract causes hepatic atrophy in transgenic models and dysregulates genes involved in bile acid transport and lipid metabolism, suggesting a mechanism linked to metabolic imbalance (Gao S. Y. et al., 2024). Even plants considered for common consumption, like *Enydra fluctuans* Lour., can induce marked hepatocyte vacuolation at high doses (Xavier and Kripasana, 2020).

**TABLE 4:** Hepatotoxicity profiling of natural products in zebrafish models.

Toxic substance	Plants sources	Model	Findings	Ref(s)
Radix *Sophorae tonkinensis* extract (RSTE) Ethanol extract; n-Butyl ethanol extract; Dichloromethane extract Dealkalized water extract	Radix *Sophorae tonkinensis*	Wild type, AB Zebrafish larvae at 3dpf	Dose-dependent hepatotoxicity: liver darkening and reduced blood flow RSTE induced liver shrinkage; n-butanol extract delayed yolk absorption	Liu et al. (2017)
70% EtOH extract anthraquinone and anthrones	*Polygonum multiflorum Thunb*	Wild type AB Zebrafish embryo	Anthraquinones and anthrones showed marked hepatotoxicity, among the 25 isoloated compounds	Yang et al. (2018)
70% EtOH extracts of PM Four fractions from macroporous resin (componen-ts A, B, C and D) 19 compounds from component D	*Polygoni Multiflori Thunb*. (PM)	Wild type AB zebrafish larvae at 72 h	Higher toxicity of component D compared to A–C Three anthraquinones, 6 anthrones, and 2 naphthols exhibited significant embryotoxicity, compared to other 27 isolated compounds	Li et al. (2020)
Distilled water extract	*Phytolacca acinosa Roxb*. (PAR)	Wild type AB zebrafish larvae at 96 h	LC_10_ = 1,503.69 μg/mL Caspase-dependent hepatotoxicity**:** ALT/AST**↑**, Caps3**↑** Hepatocyte loss, vacuolation, disorganization observed	Cao et al. (2021)
Distilled water extract	*Euphorbia kansui*	Wild type, AB Zebrafish larvae at 4dpf	LC_10_ = 68.047 μg/mL Hepatocyte vacuolization and structural alterations observed Caspase-dependent hepatotoxicity**:** ALT/AST**↑**, Caps3**↑**	Zhao et al. (2019)
Aqueous extract of *Psoralea corylifolia L*. (AEFP)	*Psoralea corylifolia L*	Tg (lfabp: EGFP) zebrafish larve at 72 hpf, administered for 3 days	AEFP displayed liver atrophy **↓:** hepatic fluorescence area and intensity; ALT, AST, γ-GT, TBA, TBIL, TC, TG, LDL-C, HDL-C, PPARα **↑:** SHP, CYP7A1, CYP8B1, BSEP, MRP2, NTCP, PPARγ, ME-1, SCD-1, LPL, CPT-1, CPT-2	Gao et al. (2024a)
Ethanolic Extracts of the Leaf of *Enydra fluctuans*	*Enydra fluctuans Lour*	Wild type AB zebrafish larvae	Severe hepatic vacuolization	Xavier and Kripasana (2020)
Distilled water extract	*Rhizoma Paridis*	Wild type, AB Adult zebrafish	Oxidative stress damage: MDA↑, SOD↓ Induced hepatic lipid metabolism disorder and energy imbalance Mitochondria identified as main toxic target of Rhizoma Paridis	Jia et al. (2020)
Hydroethanolic extract	*Acmella oleracea L*	Wild type, AB Adult zebrafish	LD_50_ (oral): 148.42 mg/kg; LC_50_ (immersion): 320 μg/L. seizures, tail tremors, loss of posture/movement, bottom resting, death (high-dose oral/immersion). Severe histopathological lesions in gills, liver, intestine, and kidney (high-dose oral/immersion)	Custodio de Souza et al. (2019)
The Aqueous extract (ExH_2_O) from *Genipa americana L*. leaves	*Genipa americana L*	Wild type, AB Adult zebrafish	Low-dose G. americana ExH_2_O exhibited hepatic necrosis and nuclear pyknosis; medium/high doses displayed sinusoidal dilation	Claro et al. (2023)
Astragalus polysaccharide (APS)	*Astragalus membranaceus*	One-month-old zebrafish	Liver inflammation: increased hepatic IL-1β and serum ALT Severe hepatic lipid accumulation: upregulated SREBP1 and FAS expression	Li et al. (2021a)
Palmitic Acid (PA)	*Palm*	One-month-old zebrafish	PA-altered microbiota indirectly leads ER stress and liver injury PA-modified microbiota enhanced PA absorption and aggravated hepatotoxicity	Ding et al. (2018)
*Tripterygium wilfordii* multiglycoside (GTW)	*Tripterygium wilfordii*	Tg (L-FABP: EGFP) and wild-type AB strain zebrafish larvae at 72hpf	GTW exposure caused a dose-dependent decrease in hepatic fluorescence area ALT and AST levels significantly elevated at 5 μg/mL After 72 h, hepatocytes showed sparse cytoplasm, nuclear atrophy, and severe vacuolation (dose-dependent). miR-122 expression markedly downregulated. Inflammation: il1β, il6, tnfα, il10, cox2, ptges↑; TGF-β↓ Apoptosis: caspase-8, caspase-9↑; Bcl-2↓ Proliferation: top2α, uhrf1↓ Liver function: alr, cyp3c1↑; cyp3a65↓	Xiu-Ying et al. (2021)
Triptolide (TP)	*Tripterygium wilfordii Hook.F*	Tg (L-FABP: EGFP) zebrafish larvae	Safe concentrations: 0.2, 0.4, 0.8 μM Total teratogenicity and liver scores decreased dose-dependently (24 h TP exposure) Liver area reduced with dose and time (48–72 h) Dose-dependent inhibition of yolk absorption LDL, TG, ALT, and AST levels increased with dose Liver necrosis observed in medium and high doses Fas expression upregulated dose-dependently Activated Fas–Caspase-8 pathway; autophagy genes (Beclin1, Atg5, Atg3, Lc3) significantly upregulated	Li et al. (2023a)
Triptolide	*Vine*	Tg (2.8lfabp: GFP) zebrafish larvae at 3dpf wild-type WIK line	Hepatocyte vacuolization, disorganization, necrosis, and reduced liver volume at 0.8 mM Downregulation in miR-122 expression	Vliegenthart et al. (2017)
Paris saponin I, II and VII	*Paris polyphylla*	Tg (L-FABP: EGFP) Zebrafish larvae at 3dpf	LC_50_: Paris saponin I = 122.2 ng/mL; II = 210.7 ng/mL; VII = 566.2 ng/mL Paris saponins I and VII significantly reduced liver fluorescence area; all three decreased fluorescence intensity Paris saponin II (45–100 ng/mL): hepatic vacuolation and necrosis observed (dose-dependent) TUNEL assay: Paris saponin I induced dose-dependent hepatocyte apoptosis Toxicity of Paris saponin I associated with altered p53 and Wnt pathway gene expression	Ni et al. (2023)
β-eudesmol, atractylodin	*Atractylodes lancea*	Tg (fabp10a: EGFP) Zebrafish embryo	β-Eudesmol and atractylodin (non-lethal and lethal) inhibited α1a and β1 gene expression Both compounds significantly downregulated fabp10a β-Eudesmol markedly reduced whole-body cortisol levels in embryos	Tshering et al. (2022)
Aloe emodin (AE)	*Rheum palmatum L*	Tg (fabp10: EGFP) zebrafish larvae at 72 h	AE lead severe hepatic necrosis, vacuolation, and disordered hepatocytes Upregulation in NF-κB/p53 inflammatory and apoptotic pathways	Quan et al. (2019)
Isoliquiritigenin (ISL)	*Licorice*	Wild type AB zebrafish and the Tg (cmcl2: EGFP), Tg (L-FABP: EGFP) and Tg (Vmat: GFP) zebrafish	LC_10_ = 16.31 mM; LC_1_ = 12.37 mM ISL induced developmental toxicity, death, and malformations Liver area and fluorescence intensity decreased dose-dependently Histology: hepatocytes showed loose contacts and large vacuoles ISL induced oxidative stress–mediated apoptosis *via* Nrf2–HO1/JNK–ERK/mitochondrial pathway	Song et al. (2020)
2″-O-Rhamnosylicariside II(SC) Sagittatoside B(SB)	*Herba Epimedii*	Wild type AB adult zebrafish	MNLC = 50 μM SC (25 μM) lead severe hepatocyte vacuolation, mild degeneration and edema, slight inflammatory infiltration SB (25 μM) lead hepatocyte necrosis, moderate edema, mild vacuolation, loose cell contacts, lymphocyte/monocyte infiltration, and Kupffer cell proliferation	Zhong et al. (2019)
Aurantio-obtusin (AO)	*Cassiae semen*	wild-type AB, Tg (L-FABP: EGFP) and Tg (mpo:GFP) zebrafish at 3dpf	AO delayed yolk absorption, caused liver enlargement, and enhanced inflammation in zebrafish larvae. mRNA levels of IL-6 and TNF-α were upregulated. AO-induced hepatotoxicity showed sex-dependent effects In females, AO activated NLRP signaling, increasing NLRP3 and Caspase-1 expression	Hu et al. (2022)

These abstract uses wild-type and transgenic zebrafish as experimental models to systematically summarize the inherent hepatotoxicity and regulatory characteristics of natural product extracts and their monomeric components. “↑” or “↓” indicates the regulatory effects of natural product extracts and their monomeric components on different indicators. “↑” indicates increase or upregulation; “↓” indicates decrease or downregulation.

For evaluating chronic exposure effects, adult zebrafish models offer distinct advantages. Studies have shown that aqueous extracts of Rhizoma Paridis induce oxidative stress and energy metabolism disorders (Jia et al., 2020), while extracts of Acmella oleracea can cause multi-organ necrosis at high doses (Custodio de Souza et al., 2019). Extracts from Genipa americana leaves, rich in phenols and flavonoids, trigger a range of injuries from nuclear pyknosis to hepatic sinusoidal dilation, implicating oxidative stress in multi-organ toxicity (Claro et al., 2023). Furthermore, long-term administration of compounds like Astragalus polysaccharides or palmitic acid can promote hepatic steatosis and chronic inflammation, the latter exacerbating toxicity through gut microbiota dysregulation (Ding et al., 2018; Li et al., 2021a).

The sensitivity of transgenic zebrafish is particularly valuable for dissecting specific toxic mechanisms. For example, Tripterygium wilfordii multiglycoside (GTW) and its principal bioactive compound, triptolide (TP), reduce hepatic fluorescence and induce apoptosis *via* Caspase-8 and Fas-dependent pathways, with observed cytoplasmic thinning and nuclear condensation (Huo et al., 2019; Li M. et al., 2023; Vliegenthart et al., 2017; Xiu-Ying et al., 2021). Paris saponins I, II, and VII similarly reduce fluorescence intensity and activate apoptosis through p53 and Wnt signaling (Ni et al., 2023), while sesquiterpenes like β-eudesmol impair hepatic function through metabolic interference (Tshering et al., 2022).

Among purified monomers, specific compound classes exhibit distinct hepatotoxic profiles. The anthraquinone aloe-emodin (AE) induces extensive necrosis by activating NF-κB and p53 pathways and upregulating pro-inflammatory genes (Quan et al., 2019). The flavonoid isoliquiritigenin (ISL) triggers mitochondria-dependent apoptosis by disrupting the balance between the protective Nrf2-HO-1 and the stress-related JNK/ERK pathways (Song et al., 2020). Certain glycosides cause severe histopathological lesions including vacuolization and necrosis in adult fish (Zhong et al., 2019), and aurantio-obtusin (AO) drives inflammation-driven hepatotoxicity by activating NLRP3 inflammasomes (Hu et al., 2022).

Thus, zebrafish models provide a comprehensive and integrative framework for hepatotoxicity assessment, effectively complementing their role in efficacy screening. They enable detection of dose-dependent effects from acute morphological changes to chronic pathological outcomes. Larval models are ideal for high-throughput acute toxicity studies, adult models for chronic exposure assessment, and transgenic lines for real-time, mechanistic investigation. This capacity for systematic safety profiling solidifies the zebrafish as an indispensable dual-purpose platform in natural product research, ensuring that the pursuit of therapeutic potential is consistently balanced with a rigorous understanding of toxicological risk, a balance essential for successful translational development.

## 6 Zebrafish models and phytochemicals: an integrated approach to liver health and disease

**FIGURE 1:**
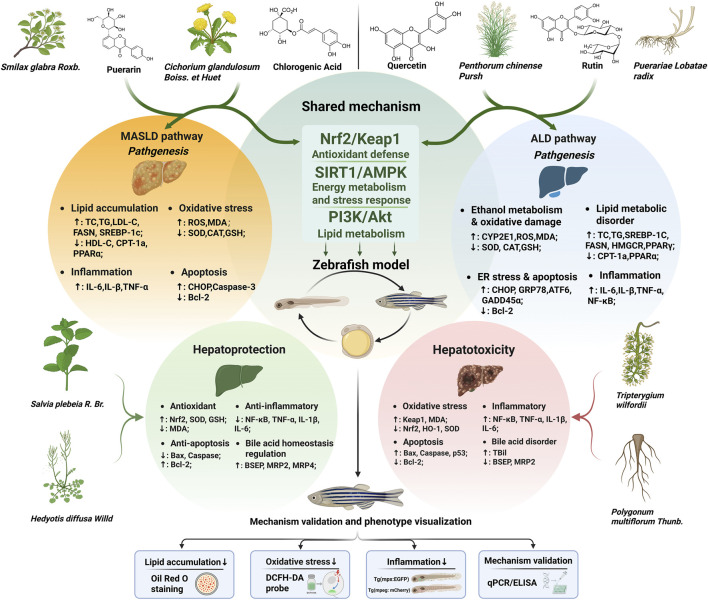
The zebrafish as a dual-purpose platform for evaluating natural products in liver disease. The schematic integrates the model’s applications in studying disease mechanisms, hepatoprotection, and hepatotoxicity. The left (orange) and right (blue) panels summarize the core pathogenic cascades—involving lipid accumulation, oxidative stress, inflammation, and apoptosis—in MASLD and ALD, respectively. The central green area highlights key molecular pathways (e.g., Nrf2/Keap1, SIRT1/AMPK, PI3K/Akt) commonly modulated by protective phytochemicals, as validated in zebrafish. The lower section contrasts the beneficial effects of representative hepatoprotective compounds (green) against the detrimental mechanisms of hepatotoxic agents (red), emphasizing how the model enables simultaneous investigation of antioxidant, anti-inflammatory, and metabolic regulation. This figure was created in BioRender (Justin, B, 2026; https://BioRender.com/3cwljbw) and is published under agreement No. HS29IK5Q97.

This review has systematically charted the application of the zebrafish model across the spectrum of liver disease research, from metabolic disorders like MASLD and ALD to chemical-induced injury, and crucially, in the parallel assessment of therapeutic efficacy and inherent hepatotoxicity of phytochemicals. As synthesized in Figure 1, the zebrafish provides a unique *in vivo* lens through which the multifaceted pathogenesis of liver diseases—encompassing lipid dysregulation, oxidative stress, inflammation, and apoptosis—can be visualized and dissected in real time. More importantly, the model has been instrumental in validating those diverse natural products, despite their varied origins, often converge on a core set of conserved protective pathways, such as Nrf2/Keap1, SIRT1/AMPK, and PI3K/Akt signaling. Conversely, it equally exposes compounds that disrupt hepatic homeostasis through inflammatory or metabolic toxicity. This dual capacity for efficacy and safety screening establishes the zebrafish not merely as a tool, but as a highly efficient and cost-effective integrative platform for the early-stage discovery and evaluation of plant-derived hepatoprotective agents (Katoch and Patial, 2021; Shimizu et al., 2023).

To fully realize this translational potential, it is essential to acknowledge and strategically address the model’s limitations. A primary consideration is the interspecies divergence in key physiological areas, including specific cytochrome P450 enzyme activities, aspects of immune system development, and liver regeneration dynamics (Abu-Siniyeh et al., 2025; Shehwana and Konu, 2019). These differences can influence the metabolic fate of compounds and the precise recapitulation of chronic human disease progression. The future of the field, therefore, lies in moving from generic modeling to precision humanization. Emerging gene-editing technologies, particularly CRISPR/Cas9, now allow for the precise introduction of human disease-associated alleles or even the replacement of zebrafish genes with their human counterparts, creating “humanized” models that promise far greater fidelity in mimicking human pathology and pharmacological response (Haberlein et al., 2022; Li et al., 2016; Schellens et al., 2022). Furthermore, while the gut-liver axis is established in liver disease pathophysiology (Anand and Mande, 2022), its integration into natural product screening remains nascent. The use of germ-free or human-microbiota-associated zebrafish, combined with multi-omics analyses, presents a transformative opportunity to unravel how phytochemicals mediate their effects through host-microbiome interactions, a dimension largely unexplored in current zebrafish-based studies (Garibay-Valdez et al., 2024; Zhong et al., 2022).

The research landscape in zebrafish hepatology, while rich, currently exhibits notable gaps. The predominant focus has been on early-stage metabolic steatosis and acute injury, with comparatively limited exploration of advanced chronic conditions such as fibrosis, viral hepatitis, autoimmune hepatitis, and hepatocellular carcinoma (Lin et al., 2025; Shimizu et al., 2023; Wang et al., 2021). This gap partly stems from the traditional reliance on larval models for speed, leaving adult zebrafish underutilized for studies of chronic exposure, cumulative toxicity, and long-term therapeutic efficacy (Choi T. Y. et al., 2021; White and Patton, 2023). Future efforts must prioritize the development and validation of robust chronic and oncogenic models in zebrafish, building on promising proofs-of-concept, such as the use of transgenic hepatocellular carcinoma models to evaluate compounds like oligo-fucoidan (Wu et al., 2020). Parallel to model expansion is the pressing need for methodological harmonization. The inherent complexity of natural product extracts, combined with substantial inter-laboratory variability in protocols—covering zebrafish strain, age, diet, and endpoint analyses—poses a significant challenge to reproducibility and data translation. Advancing the field will require a concerted move towards standardized operating procedures, augmented by automated high-throughput systems. Moreover, overcoming the current paucity of pharmacokinetic data through techniques like micro-dialysis-LC/MS/MS and physiologically based pharmacokinetic (PBPK) modeling is critical for achieving reliable cross-species dose translation and enhancing preclinical predictive value.

In addition, a fundamental challenge for the broader acceptance of zebrafish in preclinical hepatology research lies in its comparison to established mammalian models, particularly rodents. Mammalian models, despite their higher cost and lower throughput, offer advantages in terms of physiological similarity in liver architecture, drug metabolism enzyme profiles, and fibrotic progression dynamics. The zebrafish liver lacks distinct portal triads and has a less complex immune system, which may influence the recapitulation of certain chronic inflammatory processes. However, the zebrafish model should not be viewed as a replacement but rather as a complementary front-line platform. Its strengths in high-throughput screening, real-time imaging, and rapid genetic manipulation enable the efficient prioritization of candidates and mechanistic hypothesis generation, which can then be validated in mammalian systems. Recognizing this complementary role is essential for the strategic integration of zebrafish into the translational pipeline, maximizing its unique advantages while acknowledging its inherent limitations.

Moreover, addressing these multifaceted challenges—through humanized genetics, gnotobiotic systems, expanded disease modeling, and rigorous methodological standardization—is not merely an exercise in technical refinement. Collectively, these efforts are converging to transform the zebrafish from a primarily phenomenological screening tool into a predictive and integrative translational platform. The ultimate goal is to create a more holistic research pipeline where the model can simultaneously elucidate the complex pathophysiology of liver diseases, identify and validate the mechanistic basis of phytochemical efficacy, and provide early, reliable insights into pharmacokinetics and safety. By bridging these domains within a single, scalable *in vivo* system, the zebrafish platform stands to dramatically de-risk and accelerate the early-stage development of plant-derived therapeutics. It is this strategic evolution that positions the model to make its most significant contribution to hepatology.

## 7 Conclusion

In conclusion, the zebrafish model stands at a pivotal point in its application to liver disease and natural product research. Its unparalleled strengths in live imaging, genetic manipulability, and scalable screening have made it an indispensable front-line platform for uncovering therapeutic mechanisms and flagging toxicological risks. The path forward lies not in using the model in isolation, but in its strategic integration with next-generation technologies: humanized genetics for precision, gnotobiotic systems for ecological context, omics for holistic profiling, and advanced analytics for pharmacokinetics and standardization. By embracing this integrated, methodologically rigorous approach, the zebrafish model can transcend its current role as a screening tool and fully evolve into a predictive translational bridge. This evolution will significantly accelerate the journey of plant-derived compounds from phenomenological discovery to mechanism-informed drug candidates, directly contributing to the urgent quest for new treatment options in liver disease.

## Glossary

ACC: Acetyl CoA Carboxylase
AchE: Acetylcholinesterase
ADH: Alcohol Dehydrogenase
ALB: Albumin
ALD: Alcohol-related Liver Disease
ALT: Alanine Aminotransferase
AMPK: AMP-Activated Protein Kinase
APAP: Acetaminophen (Paracetamol)
AST: Aspartate Aminotransferase
ATP: Adenosine Triphosphate
Bax: Bcl-2–Associated X protein
Bcl-2: B-Cell Lymphoma 2
BIP: Binding Immunoglobulin Protein
CAT: Catalase
CYP: Cytochrome P450
CYP-2E1: Cytochrome P450 Family 2 Subfamily E Member 1
CCNE1: Cyclin E1
COX4: Cytochrome c Oxidase Subunit IV
CPT-1: Carnitine Palmitoyl Transferase-1
dpf: Days Post Fertilization
DsRed: Discosoma Red Fluorescent Protein
DILI: Drug-Induced Liver Injury
DRP1: Dynamin-Related Protein 1
EGFP: Enhanced Green Fluorescent Protein
ER: Endoplasmic Reticulum
FAS: Fatty Acid Synthase
FAO: Fatty Acid Oxidation
GSH: Glutathione
GSH-Px: Glutathione Peroxidase
GSR: Glutathione Reductase
GST: Glutathione-S-Transferase
γ-GT: Gamma-Glutamyl Transferase
HCD: High-Cholesterol Diet
HCF: High-Cholesterol + High-Fructose Diet
HFD: High-Fat Diet
HFrD: High-Fructose Diet
HO-1: Heme Oxygenase-1
HMGCR: 3-Hydroxy-3-Methylglutaryl-CoA Reductase
hpf: Hours Post Fertilization
IFN-γ: Interferon-γ
IL-1β: Interleukin-1 Beta
IL-6: Interleukin-6
IL-10: Interleukin-10
INH: Isoniazid
IAA: Iodoacetamide
LDL-C: Low Density Lipoprotein Cholesterol
LC3-II: Microtubule Associated Protein 1A/1B-Light Chain 3 II
**LC**_**50**_: Lethal Concentration 50%
MASLD: Metabolic dysfunction-associated steatotic liver disease
MAPK: Mitogen Activated Protein Kinase
MDA: Malondialdehyde
MFN2: Mitofusin 2
MPO: Myeloperoxidase
MRP2/4: Multidrug Resistance-Associated Protein 2/4
NAFLD: Non Alcoholic Fatty Liver Disease
NASH: Non Alcoholic Steatohepatitis
NADP/NADPH: Nicotinamide Adenine Dinucleotide Phosphate
NF-κB: Nuclear Factor Kappa-B
NO: Nitric Oxide
NQO1: NAD(P)H Quinone Oxidoreductase-1
NRF1: Nuclear Respiratory Factor 1
Nrf2: Nuclear Factor Erythroid 2-Related Factor 2
OF: Overfeeding
PPAR: Peroxisome Proliferator-Activated Receptor
PI3K: Phosphoinositide 3-Kinase
PINK1: PTEN-Induced Putative Kinase 1
ROS: Reactive Oxygen Species
RXR: Retinoid X Receptor
SOD: Superoxide Dismutase
STAT3: Signal Transducer and Activator of Transcription 3
TC: Total Cholesterol
TG: Triglycerides
TAA: Thioacetamide
TBil: Total Bilirubin
T-AOC: Total Antioxidant Capacity
TNF-α: Tumor Necrosis Factor Alpha
UCP2: Uncoupling Protein-2
UPR: Unfolded Protein Response
